# Fibroid Tumor of the Uterus

**Published:** 1893-04

**Authors:** E. T. Adams

**Affiliations:** Toronto, Can.


### FIBROID TUMOR OF THE UTERUS.

E. T. Adams, M. D., Toronto, Can.

Mr. President and Members :—I have briefly to mention some peculiarities occurring in the case of a patient suffering from fibroid tumor of the uterus, and which I reported *in extenso* at our last meeting.

Perhaps some of you will remember the very satisfactory results obtained by the use of Lil-tig.$^{cm}$ in checking the profuse floodings. Monthly discharge became as natural as for many years—every three weeks and lasting four days. The tumor ceased to grow—then began to diminish and continued to diminish. The woman became natural and normal in her mental condition. In fact, patient like, a common remark with her was : "I doubt whether I ever had a tumor ;" and as improvement went on "I don't believe I ever had a tumor." Such was the happy state of affairs till the middle of November last, when one midnight a messenger delivered a telegram stating that an only and much loved sister had died very suddenly. The shock from the sudden news and grief at its cause affected her very severely. In three days you would scarcely have recognized the woman. She returned from the funeral and in about two week called on me.

She was in a most despondent state, similar to that of the year before. Thought she was going mad—was confident people would notice that there was something wrong with her mentally. Was sure some fearful misfortune was about to happen. Was confused, forgetful, no life or energy, no inclination for business—love of which, when well, was as the breath in her nostrils—anxious and given to spells of weeping. Was much bloated, and felt sure that the tumor was again growing. I gave her Lil-tig.$^{cm}$ again, and at the end of two weeks found but little improvement—possibly a slight relief mentally. At this visit she was sure the tumor was again growing, and she was confident the growth now was on the opposite side of womb. On examination this proved correct. The tumor was rapidly developing, while on the side originally affected, I could discover no enlargement. At this time she also complained of numbers of small warts over her upper abdomen, and on upper left chest and shoulder. Lil-tig. had evidently done all it could, and after careful comparison, I gave Calc-carb.$^{2c}$, three doses, two hours apart, and Placebo.

In a couple of weeks she returned and mentally was much better, brighter, and more cheerful, with desire to attend to her business returning. Quite a satisfactory change, and she received a supply of Placebo.

The improvement continued gradually and all round. Two weeks later she felt sure that growth in tumor had ceased, and she began again to expect to be a well woman. Some time in February improvement seemed to cease, after a severe cold. By the way, disposition to take cold easily was a prominent condition in the beginning of this her second attack, and I gave her Calc-carb.$^{cm}$, one dose. This was the last active medicine she received. She has been comparatively well all the past spring, and as she begins to "wonder whether she had a tumor at all," I regard her prospects as favorable though uncertain, but that this may prove a perennial root and thus afford me an annual branch on which to address you.

To me this case has been very peculiar. No doubt could exist as to the presenee of a tumor in 1890 and early part of 1891. It was so diagnosed by several gynecologists—the profuse hemorrhages, etc.

And no doubt can exist that she was on the road to health if not cured prior to the shock, grief, etc. Then the sudden return, and on the opposite side of womb. I cannot account for this. It's peculiar.

But one thing prevents my hoping too much from past success in this case, and that is that rheumatism (muscular), from which she has suffered for years, has not been to any appreciable extent even relieved by the treatment.

On the other hand, she keeps a medicine chest with remedies in low potencies with which she doses herself for cold and slight attacks, only sending for the doctor when either she or her friends get alarmed. But the drugs she took did not in any way seem to interfere with the action of the CM potency. Possibly they did with the 2c.

I neglected to say that the crop of warts is disappearing rapidly.

### Discussion.

Dr. Johnson—One point Dr. Adams brought out which I think is important, namely, that remedies in lower potencies do not affect the action of a higher potency. I have noticed it in several cases. When a patient who is under the action of a constitutional remedy has an acute attack we may give an appropriate remedy in a lower potency without affecting or disturbing the action of the higher.

Dr. W. L. Morgan—I have observed the same thing, that the lower potencies do not interfere with the higher. This case reminds me of one I had five years ago, a fibroid tumor. The patient had had a large one taken away nine years before, and this was still larger than the one taken away. It was so large that when it came down in the pelvis I could not get my finger in the vagina above it. It interfered with the flow of urine. It was exceedingly hard and very tender to touch. Her symptoms called for Sepia. I gave the 200th. In three months it removed the entire tumor, and it remained absent for a long time. Returning again in six months Sepia$^{1m}$ removed it again. An old laceration of the cervix only remained, until last fall she went into a hospital and had an operation. After that came prolapse without return of tumor, for which she came to my office a few days ago. She had been wearing a pessary. I gave her Sepia$^{cm}$, and have not seen her since. That was just before leaving home for this place. She was much improved two months later.

